# Changes in alcohol consumption and the risk of postmenopausal breast cancer in the European Prospective Investigation into Cancer and Nutrition cohort

**DOI:** 10.1007/s00394-026-04008-5

**Published:** 2026-06-19

**Authors:** Christian S. Antoniussen, Daniel B. Ibsen, Anja Olsen, Kim Overvad, Fanélie Vasson, Gianluca Severi, Dzevka Dragic, Thérèse Truong, Renée T. Fortner, Charlotte Le Cornet, Matthias B. Schulze, Chiara Di Girolamo, Valeria Pala, Chiara Doccioli, Antonio Agudo, Marcela Guevara, Sandar Tin Tin, Isobel G. Jackson, Marc J. Gunter, Laure Dossus, Pietro Ferrari, Christina C. Dahm

**Affiliations:** 1 Department of Public Health, Aarhus University, Bartholins Allé 2, 8000 Aarhus, Denmark; 2 Steno Diabetes Center Aarhus, Aarhus University Hospital, Aarhus, Denmark; 3 Department of Nutrition, Exercise and Sports, University of Copenhagen, Copenhagen, Denmark; 4 Danish Cancer Institute, Copenhagen, Denmark; 5 Nutrition and Metabolism Branch, International Agency for Research on Cancer (IARC), World Health Organization, Lyon, France; 6 UVSQ, Inserm, Gustave Roussy, CESP, Paris-Saclay University, Villejuif, Paris, France; 7 Department of Statistics, Computer Science and Applications “G. Parenti”, University of Florence, Florence, Italy; 8 Division of Cancer Epidemiology, German Cancer Research Center, Heidelberg, Germany; 9 Department of Research, Cancer Registry of Norway, Norwegian Institute of Public Health, Oslo, Norway; 10 Department of Molecular Epidemiology, German Institute of Human Nutrition, Potsdam-Rehbruecke, Nuthetal, Germany; 11 Institute of Nutritional Science, University of Potsdam, Nuthetal, Germany; 12 Department of Clinical and Biological Sciences, University of Turin, Regione Gonzole, 10, 40134 Orbassano, TO Italy; 13 Epidemiology and Prevention Unit, Fondazione IRCCS Istituto Nazionale Dei Tumori Di Milano, Milan, Italy; 14 Clinical Epidemiology Unit, Institute for Cancer Research, Prevention and Clinical Network (ISPRO), Florence, Italy; 15 Unit of Nutrition and Cancer, Catalan Institute of Oncology - ICO, L’Hospitalet de Llobregat, Barcelona, Spain; 16 Nutrition and Cancer Group, Bellvitge Biomedical Research Institute - IDIBELL, L’Hospitalet de Llobregat, Barcelona, Spain; 17 Instituto de Salud Pública y Laboral de Navarra, 31003 Pamplona, Spain; 18 Centro de Investigación Biomédica en Red de Epidemiología y Salud Pública (CIBERESP), 28029 Madrid, Spain; 19 Navarra Institute for Health Research (IdiSNA), 31008 Pamplona, Spain; 20 Cancer Epidemiology Unit, Oxford Population Health, University of Oxford, Oxford, UK; 21 Imperial College London, London, UK

**Keywords:** Alcohol consumption changes, Breast cancer, European Prospective Investigation into Cancer and Nutrition, Hormonal receptor status

## Abstract

**Purpose:**

Alcohol consumption is a cause of breast cancer (BC), yet the association between changes in alcohol consumption during adulthood and the risk of BC has been examined little. This study aimed to investigate the association between midlife changes in alcohol consumption and the risk of BC.

**Methods:**

Within the European Prospective Investigation into Cancer and Nutrition cohort including 123,679 women, changes in alcohol intake were obtained by comparing middle-aged participants’ alcohol intake assessed at recruitment and during follow-up, 9.8 years (median) later. Missing information about follow-up alcohol intake and covariates was multiple imputed. In the primary analysis, changes in alcohol consumption were investigated continuously as a change in alcohol intake of 10 g/day, calculated by subtracting the baseline intake (g/day) from the follow-up intake (g/day) and divided by 10 in relation to the risk of subsequent postmenopausal BC, overall, and by hormonal receptor status: estrogen receptor (ER), progesterone receptor (PR), and human epidermal growth factor receptor 2 (HER2). In a secondary analysis, changes in alcohol intake were categorized in nine combinations of three intake groups at baseline and follow-up (≤1 g/day, >1–8 g/day, and >8 g/day)., Multivariable Cox proportional hazards regression models were used to estimate hazard ratios (HRs) with 95% confidence intervals (CIs).

**Results:**

During a median follow-up time of 4.0 years after the follow-up assessment, 2,173 cases of postmenopausal BC were diagnosed. No associations were observed between alcohol changes and BC risk (HR: 0.97, 95% CI 0.93, 1.01) per 10 g/day nor with ER−/PR−, ER+/PR, ER+/PR+, HER2−, or HER2+ specific BC.

**Conclusion:**

Changes in alcohol consumption during midlife were not associated with the risk of postmenopausal BC, either overall or by hormonal receptor status.

**Supplementary Information:**

The online version contains supplementary material available at 10.1007/s00394-026-04008-5.

## Background

Consumption of alcoholic beverages is considered to be a cause of invasive female breast cancer (BC) by large cancer research institutions [1, 2]. According to the Global Cancer Observatory by the International Agency for Research on Cancer (IARC), BC is the most diagnosed cancer disease among women worldwide with the number of cases expected to increase the coming years [3]. Recently, more than 98,000 (95% uncertainty intervals: 68,200–130,500) BC cases worldwide were estimated to be attributed to alcohol drinking [4, 5].

The adult per capita consumption of alcohol increased from 5.9 L (95% CI 5.8–6.1) in 1990 to 6.5 L (95% CI 6.0–6.9) in 2017 worldwide, and a further increase of 17% by 2030 is expected while the number of lifetime abstainers is expected to decrease [6]. Although the average alcohol intake in Europe has decreased the last decades, the World Health Organization (WHO) European region had the highest alcohol consumption per capita in 2019 [6]. Consumption of alcoholic beverages is an important target for primary prevention of BC. In 2024, the WHO published its global action plan 2022–2030 intending to roll out a Global Strategy that includes the WHO “best buys” recommendations to reduce the consumption of alcoholic beverages in order to mitigate the harmful effects of alcohol consumption on health outcomes, including cancer [7]. Nevertheless, the current knowledge of whether changes in alcohol consumption during adulthood are associated with the incidence of BC remains limited [8, 9].

Two studies have investigated associations between changes in alcohol consumption assessed longitudinally and the risk of BC. A large Korean cohort study of more than 2 million women operationalized changes in alcohol consumption between two alcohol assessments two years apart using 16 combinations of intake. In that study, an intake of ≥30 g/day that was reduced to an intake of 15.0–29.9 g/day was associated with a lower risk of BC compared to a consistently high intake [10]. A similar result was found for an alcohol intake that was reduced from a low alcohol intake (<15 g/day) to none (0 g/day) compared to a consistent low intake. No association with BC was found for an increased alcohol intake. In a Danish prospective study of 5-year changes in alcohol consumption and the risk of BC, an increased alcohol intake was associated with a higher risk of postmenopausal BC compared to a stable intake. Yet, no association with BC was found for a decreased alcohol intake compared to a stable intake [11]. The IARC Handbook of Cancer Prevention on reducing or ceasing alcohol consumption recently concluded that only limited evidence exists that cessation lowers the risk of BC [9]. In this study, we investigated whether changes in alcohol intake in midlife were associated with the risk of postmenopausal BC and its hormonal-related subtypes in the European Prospective Investigation into Cancer and Nutrition (EPIC) cohort, using longitudinal data on alcohol consumption from six different European countries.

## Material and methods

### Study population

EPIC is a prospective multicenter study of 521,323 participants aged 25–70 years at recruitment. Participants were recruited between 1992 and 2000 across 23 study centers within 10 European countries, i.e. United Kingdom, France, Germany, Norway, Sweden, the Netherlands, Denmark, Italy, Greece, and Spain [12, 13]. At baseline, all participants filled in validated country or study center-specific self-administered dietary and lifestyle questionnaires. In this study, after a median of 9.9 years (IQR: 5.2–12.0) from recruitment, participants filled in follow-up lifestyle questionnaires comparable to the questionnaires completed at recruitment.

Data from Greece were unavailable for administrative reasons (n = 28,561). We further excluded all male participants (n = 141,478), women with prevalent cancer at the time of recruitment (n = 20,741), and those for whom we had no follow-up time after recruitment (n = 2,195), no baseline information (n = 3,237), and those in the top and bottom 1% of the ratio of baseline energy intake to estimated energy requirements (n = 6,425).

**Fig. 1:**
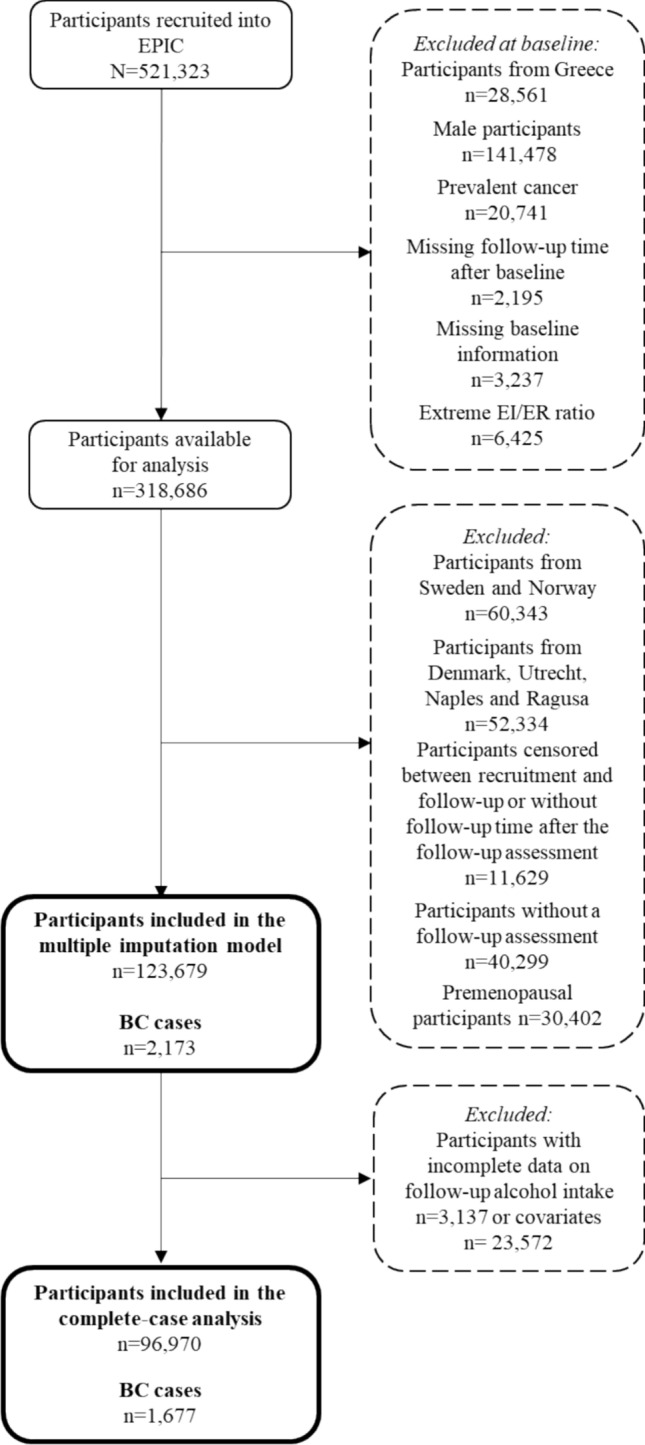
Inclusion of participants into the study

We also excluded participants from Sweden and Norway due to administrative reasons (n = 60,343), and participants from Denmark, Utrecht (the Netherlands), Naples and Ragusa (Italy) where information about participants’ alcohol intake during follow-up was not centralized in EPIC (n = 52,344). We also excluded participants who were censored between the baseline and the follow-up assessments, including those diagnosed with cancer, and those who did not have follow-up time after the follow-up assessment (n = 11,629) including participants without a follow-up assessment (n = 40,299), as detailed in Online Resource, Supplementary Table S1. Online Resource, Supplementary Table S2 compares their baseline characteristics with participants included in the study. Finally, we excluded women who were premenopausal at follow-up (n = 30,402). To investigate associations between changes in alcohol consumption and risk of BC, multiple imputation by chained equations (MICE) were used to impute missing data on follow-up information about alcohol intake and potential confounders for the final set of data (n = 123,679, n, cases: 2,173). A complete case analysis on 96,970 participants (cases, n = 1,677) was also conducted after the exclusion of 26,709 (approximately 21.6%) participants with missing alcohol data from the follow-up assessment (n = 3,137) and covariates (n = 23,572), respectively as detailed in Fig. 1**.**

### Changes in alcohol intake

At recruitment and during follow-up, all participants reported their average intake of standard drinks of wine, fortified wine, beer/cider, and spirits/liquor over the last 12 months on dietary or lifestyle questionnaires [13]. The total alcohol intake was calculated in g/day in each country using information about beverage-specific average glass volumes obtained from 24-h dietary recall interviews of a subgroup of the EPIC cohort [14, 15]. Online Resource, Supplementary Table S3 shows the country-specific proportions of participants with information about their alcohol intake at baseline and follow-up. The primary exposure was operationalised as change in alcohol intake of approximately 1 standard drink/day (10 g/day) calculated by subtracting the baseline intake (g/day) from the follow-up intake (g/day) and divided by 10 [16]. For descriptive purposes we grouped the participants into categories of alcohol intake changes between baseline and follow-up: “decreased intake” corresponding to <−8 g/day, or −8 to <−1 g/day,” stable alcohol intake” corresponding to −1 to 1 g/day, and “increased” alcohol intake (>1 to 8 g/day) or (>8 g/day). Changes were further categorized in nine combinations of three intake groups at baseline and follow-up (≤1 g/day, >1 to 8 g/day, and >8 g/day), i.e. ≤1 g/day at both baseline and follow-up; ≤1 g/day at baseline and >1 to 8 g/day at follow up; etc. We chose the categorisation given the distribution of alcohol intake within the study population with median alcohol intakes at baseline and follow-up assessments of 4.1 g/day (10th percentile–90th percentile: 0.0–24.2) and 4.3 g/day (10th percentile–90th percentile: 0.0–25.1), respectively. The median follow-up alcohol intake including the 10th and 90th percentiles were calculated after exclusion of participants with missing information about their follow-up alcohol intake (n = 3,137).

### Ascertainment of breast cancer

BC cases were identified through record linkage to population-based cancer registries in the Netherlands, Italy, the United Kingdom, and Spain. In France and Germany information about BC diagnosis was obtained from either pathology reports, cancer registries, insurance records, or active follow-up procedures by contacting study participants or their next-of-kin [13]. BC was defined as invasive BC (C-50) according to the International Classification of Disease for Oncology. Information about estrogen receptor (ER), progesterone receptor (PR), and human epidermal growth factor (HER2) was obtained from pathology reports in 20 EPIC centers. In EPIC, a positive receptor status was determined based on the following criteria as described previously: ≥10% cells stained, any “plus-system” description, ≥20 fmol/mg, an Allred score of ≥3, an IRS of ≥2, or an H-score ≥ 10 [17].

### Covariate assessment and menopausal status definition

From lifestyle questionnaires administered at baseline, we obtained information about number of full-term pregnancies, age at first full-term pregnancy, ever use of hormonal replacement therapy, educational level, smoking status, and physical activity level. Anthropometry information was self-reported in the Oxford study center (UK) and France, and measured in the remaining countries [13]. Body mass index (BMI) was calculated as weight (kg) divided by height squared (m^2^). Anthropometric information was standardized in EPIC [13].

We used follow-up information about menopausal status collected simultaneously with information about follow-up alcohol intake to determine the participants’ menopausal status according to their menstrual history [18, 19]. Participants who reported not having menstrual bleeding in the preceding 12 months or those reporting bilateral oophorectomy were categorized as postmenopausal. Women who had <9 menstruation cycles the past year or stated they had menstruated in the past 12 months but had stopped menstruating at the time of the assessment were classified as perimenopausal. For participants with no available information about their menstruation history, participants reporting use of oral contraceptives or hormonal replacement therapy or women who had undergone surgical hysterectomy, their menopausal status was determined based on information about their age at the follow-up assessment. Women ≥ 55 years old were categorised as postmenopausal and perimenopausal if they were aged 46–55 years [18, 19]. We classified perimenopausal women (n = 15,026) as postmenopausal under the assumption that they would have reached menopause by the time of BC diagnosis.

### Statistical analysis

**Fig. 2:**
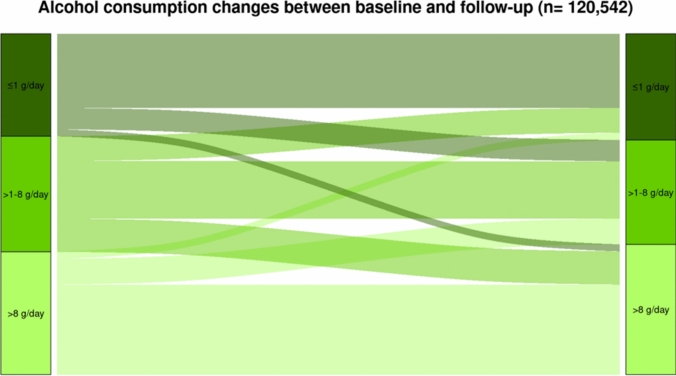
Sankey plot of categories of alcohol consumption from baseline through follow-up assessments among participants with complete information about their alcohol intake (120,542)

Summary statistics were used to present baseline characteristics (Table 1). We grouped all participants with complete alcohol information across the baseline and follow-up assessments in categories of: ≤1 g/day, >1 to 8 g/day, and >8 g/day, separately at baseline and during follow-up, to visualise 9 possible transitions in alcohol intake using a Sankey diagram **(**Fig. 2).

**Table 1:** Characteristics of the study population according to change in alcohol consumption between the baseline and follow-up assessment (n = 123,679)

Change in intake of alcohol (g/day) between baseline and follow-up (n = 123,679)						
	Decreased intake		Stable intake	Increased intake		Participants with missing information about follow-up alcohol intake
Characteristics^1^	<−8	>−8 to <−1	−1 to 1	>1 to 8	>8	Missing^2^
Number of participants, n (%)	13,958 (11.4)	26,101 (21.1)	37,755 (30.5)	26,330 (21.3)	16,398 (13.3)	3,137 (2.5)
Number of BC cases, n (%)	241 (1.7)	458 (1.8)	691 (1.8)	484 (1.8)	263 (1.6)	36 (1.1)
Estrogen receptor status (ER), n (%)†						
ER−	26 (10.8)	67 (14.6)	75 (10.9)	67 (13.8)	29 (11.0)	NR^3^
ER+	178 (73.9)	295 (64.4)	488 (70.6)	321 (66.3)	195 (74.1)	26 (72.2)
Missing*	37 (15.4)	96 (21.0)	128 (18.5)	96 (19.8)	39 (14.8)	NR^3^
Progesterone receptor status (PR), n (%)^†^						
PR−	52 (21.6)	115 (25.1)	138 (20.0)	115 (23.8)	52 (19.8)	10 (27.8)
PR+	138 (57.3)	207 (45.2)	353 (51.1)	199 (41.1)	127 (48.3)	19 (52.8)
Missing*	51 (21.2)	136 (29.7)	200 (28.9)	170 (35.1)	84 (31.9)	7 (19.4)
Human epidermal growth factor receptor 2 (HER2), n (%)^†^						
HER2−	144 (59.8)	240 (52.4)	360 (52.1)	250 (51.7)	145 (55.1)	20 (55.6)
HER2+	27 (11.2)	54 (11.8)	93 (13.5)	51 (10.5)	30 (11.4)	7 (19.4)
Missing*	70 (29.0)	164 (35.8)	238 (34.4)	183 (37.8)	88 (33.5)	9 (25.0)
Age at baseline (y), median (p10–p90)	51.8 (44.0, 62.1)	52.3 (44.0, 62.8)	53.2 (44.3, 63.6)	51.3 (44.0, 62.7)	51.5 (44.2, 62.9)	51.9 (41.5, 64.1)
*Missing*	0.0 (0)	0.0 (0)	0.0 (0)	0.0 (0)	0.0 (0)	0.0 (0)
Age at follow-up (y), median (p10–p90)	61.3 (51.3, 72.0)	60.8 (50.7, 71.8)	61.1 (50.9, 71.9)	60.7 (51.4, 71.8)	61.5 (52.7, 72.8)	63.0 (52.2, 75.5)
*Missing*	0.0 (0)	0.0 (0)	0.0 (0)	0.0 (0)	0.0 (0)	0.0 (0)
Intake of alcohol at baseline (g/day), median (p10–p90)	24.2 (12.0, 50.5)	7.0 (1.9, 22.1)	0.4 (0.0, 6.0)	3.4 (0.0, 15.3)	7.2 (0.5, 24.0)	2.0 (0.0, 24.2)
*Missing*	0.0 (0)	0.0 (0)	0.0 (0)	0.0 (0)	0.0 (0)	0.0 (0)
Intake of alcohol at follow-up (g/day), median (p10–p90)	7.7 (0.0, 25.9)	3.0 (0.0, 17.3)	0.0 (0.0, 6.1)	7.8 (2.2, 19.9)	23.9 (12.1, 50.6)	
*Missing*	–	–	–	–	–	–
Educational level, n (%)						
None	619 (4.4)	1,119 (4.3)	4,841 (12.8)	827 (3.1)	156 (1.0)	45 (1.4)
Primary	3,090 (22.1)	5,568 (21.3)	9,838 (26.1)	4,043 (15.4)	2,277 (13.9)	1,463 (46.6)
Technical/professional school	1,790 (12.8)	4,349 (16.7)	5,191 (13.7)	3,870 (14.7)	2,090 (12.7)	529 (16.9)
Secondary school	3,932 (28.2)	6,619 (25.4)	8,089 (21.4)	7,763 (29.5)	5,373 (32.8)	675 (21.5)
University degree	4,077 (29.2)	6,900 (26.4)	7,337 (19.4)	7,727 (29.3)	5,233 (31.9)	348 (11.1)
* Missing*	450 (3.2)	1,546 (5.9)	2,459 (6.5)	2,100 (8.0)	1,269 (7.7)	77 (2.5)
BMI, n (%)						
<18.5 kg/m^2^	232 (1.7)	445 (1.7)	843 (2.2)	652 (2.5)	429 (2.6)	42 (1.3)
18.5–24.9 kg/m^2^	8,314 (59.6)	15,160 (58.1)	18,405 (48.7)	16,928 (64.3)	11,237 (68.5)	1,510 (48.1)
25–<30 kg/m^2^	3,967 (28.4)	7,463 (28.6)	11,494 (30.4)	6,609 (25.1)	3,747 (22.9)	1,121 (35.7)
≥30 kg/m^2^	1,445 (10.4)	3,033 (11.6)	7,013 (18.6)	2,141 (8.1)	985 (6.0)	464 (14.8)
* Missing*	0 (0.0)	0 (0.0)	0 (0.0)	0 (0.0)	0 (0.0)	0 (0.0)
Smoking status, n (%)						
Never	7,884 (56.5)	16,645 (63.8)	27,037 (71.6)	16,746 (63.6)	9,117 (55.6)	1,948 (62.1)
Former	3,314 (23.7)	5,681 (21.8)	6,155 (16.3)	6,195 (23.5)	4,484 (27.3)	583 (18.6)
Current	2,400 (17.2)	3,146 (12.1)	3,842 (10.2)	2,599 (9.9)	2,162 (13.2)	565 (18.0)
* Missing*	360 (2.6)	629 (2.4)	721 (1.9)	790 (3.0)	635 (3.9)	41 (1.3)
Physical activity level, n (%)						
Inactive	3,157 (22.6)	5762 (22.1)	11,660 (30.9)	5,189 (19.7)	3,040 (18.5)	719 (22.9)
Moderately inactive	5,680 (40.7)	10,339 (39.6)	14,092 (37.3)	10,041 (38.1)	6,225 (38.0)	1261 (40.2)
Moderately active	3,374 (24.2)	6,715 (25.7)	7,961 (21.1)	7,509 (28.5)	4,669 (28.5)	629 (20.1)
Active	1,655 (11.9)	3,042 (11.7)	3,594 (9.5)	3,189 (12.1)	2,173 (13.3)	424 (13.5)
* Missing*	92 (0.7)	243 (0.9)	448 (1.2)	402 (1.5)	291 (1.8)	104 (3.3)
Number of full-term pregnancies/age at first full-term pregnancy, n (%)						
No full-term pregnancy	1,771 (12.7)	3,203 (12.3)	4,366 (11.6)	2,954 (11.2)	1,852 (11.3)	310 (9.9)
1, <30 years	1,886 (13.5)	3,303 (12.7)	4,155 (11.0)	2,643 (10.0)	1,647 (10.0)	360 (11.5)
1, ≥30 years	778 (5.6)	1,274 (4.9)	1,811 (4.8)	1,258 (4.8)	732 (4.5)	153 (4.9)
2, <30 years	4,858 (34.8)	9,256 (35.5)	12,401 (32.8)	9,267 (35.2)	5,835 (35.6)	1,012 (32.3)
2, ≥30 years	655 (4.7)	1,244 (4.8)	1,791 (4.7)	1,414 (5.4)	843 (5.1)	149 (4.7)
≥3, <30 years	3,088 (22.1)	6,086 (23.3)	10,682 (28.3)	6,384 (24.2)	3,919 (23.9)	627 (20.0)
≥3, ≥30 years	161 (1.2)	355 (1.4)	642 (1.7)	417 (1.6)	224 (1.4)	34 (1.1)
* Missing*	761 (5.5)	1,380 (5.3)	1,907 (5.1)	1,993 (7.6)	1,346 (8.2)	492 (15.7)
Ever use of hormonal replacement therapy, n (%)						
Yes	4,143 (29.7)	8,072 (30.9)	9,868 (26.1)	7,777 (29.5)	5,175 (31.6)	554 (17.7)
No	8,816 (63.2)	15,776 (60.4)	25,747 (68.2)	17,081 (64.9)	10,587 (64.6)	2,513 (80.1)
* Missing*	999 (7.2)	2,253 (8.6)	2,140 (5.7)	1,472 (5.6)	636 (3.9)	70 (2.2)

N/n: Number, p: Percentile, %: Percentage, BC; Breast Cancer, BMI: Body Mass Index, y: Years, g/day: gram per day, –: not applicable, NR: not reported

^†^Numbers cover cases of BC

*Cases with missing information about hormonal receptor status were censored in the Cox regression models of hormonal subtypes

^1^Numbers are rounded to one decimal place

^2^Characteristics of participants with missing information about their alcohol intake at follow-up

^3^Numbers below 5 are not reported to comply with the GDPR regulations

### Multiple imputation

MICE was used to impute missing values for follow-up alcohol intake (n = 3137) and covariates measured at baseline (n = 23,572) under the assumption of missing at random [20]. Alcohol intake at follow-up (continuous) and covariates considered potential confounders were included in the models: education level (categorical), smoking status (categorical), physical activity level (categorical), number of full-term pregnancies/age at first full-term pregnancy (categorical), and ever use of hormonal replacement therapy (dichotomous). We also included alcohol intake at baseline (continuous), EPIC study center (categorical), age at the baseline assessment (continuous), age at the follow-up assessment (continuous), the breast cancer indicator (dichotomous), the time between questionnaire administration (log-transformed) (continuous) and the Nelson-Aalen cumulative hazard estimator (continuous). We imputed 25 datasets with 20 iterations for each dataset. For categorical variables, we evaluated the imputed data by comparing the distribution of the variables between the complete data and the imputed data. For the continuous measure of follow-up alcohol intake, we used a density plot. The number of imputed datasets was determined based on a calculation of the percentages of incomplete case data in terms of potential confounders as recommended by White et al. [20]. The convergence of the MICE algorithm was assessed by visual inspection of trace plots for each variable with missing information. We considered the models using the multiply imputed data as our main analyses.

### Hazard ratio estimation

Cox proportional hazards models with participants’ age as the underlying timescale were used to estimate HRs and corresponding 95% CIs for the association between changes in alcohol consumption and risk of BC. Participants were at risk of BC from their age at follow-assessment until the age of BC diagnosis, other first primary cancer (except non-melanoma skin cancer), death, emigration, loss to follow-up, or end of follow-up, whichever occurred first. In the analysis of continuous changes in alcohol consumption, potential non-linear associations between continuous change in alcohol intake and risk of BC were investigated using a restricted cubic spline with 3 knots placed at the 10th, 50th, and 90th percentiles of the difference in alcohol intake (−9.6 g/day, 0.0 g/day and 10.5 g/day, respectively). The number of knots was determined based on a comparison of the model fit of models with 3, 4, and 5 knots, respectively, using the Akaike Information Criterion with lower Akaike Information Criterion values indicating a better model fit (17). We used a likelihood ratio test to compare the log-likelihood of a model assuming non-linearity and a model assuming linearity. We investigated overall BC and hormonal receptor-specific BC in the following combinations: ER−/PR−, ER+/PR−, ER+/PR+, HER2−, and HER2+. Information on ER, PR, or HER2 was available for around 81.7%, 70.2%, and 65.4%, respectively, of all BC cases (n = 2,173). Each combination was modelled separately, by censoring participants with other hormonal receptor combinations and those with missing hormonal receptor status information. We modelled associations between all possible changes in categories of alcohol intake between baseline and follow-up and risk of BC using a stable intake within each intake category as the reference group (i.e. an intake of ≤1 g/day at both baseline and follow-up was the reference group for changes from ≤1 g/day to >1 to 8 g/day and for changes from ≤1 to >8 g/day). In analyses of change in alcohol intake as categorical variables, we investigated overall BC. For the main analyses, we fitted Cox proportional hazards models on each imputed dataset separately and estimated summary HRs with 95% CIs using Rubin’s rules [20].

We stratified all models by age at follow-up (in 1-year categories) and study center to allow for different underlying baseline hazards (Model 1). We accounted for potential confounders assessed at baseline: educational level (none, primary, technical or professional school, secondary school, university degree), BMI (<18.5 kg/m^2^, 18.5–24.9 kg/m^2^, 25–<30 kg/m^2^, ≥30 kg/m^2^), smoking status (current, former, never), physical activity level (inactive, moderately inactive, moderately active, active), number of full-term pregnancies/ age at first full-term pregnancy (No full-term pregnancy; 1, <30 years; 1, ≥30 years; 2, <30 years; 2, ≥30 years; ≥3, <30 years; ≥3, ≥30 years) and ever use of hormonal replacement therapy (yes, no) (Model 2). These variables were selected a priori based on previous literature using a directed acyclic graph (Online Resource, Supplementary Fig. S1) [21]. The proportional hazards assumption of the exposure was investigated by visual inspection of the Schoenfeld residuals [22].

### Sensitivity analyses

We conducted several sensitivity analyses to test the robustness of the results of the main analysis and overall BC*.* To assess whether the association between a change in alcohol consumption and the risk of BC was different based on the level of baseline consumption, the fully adjusted model (Model 2) was evaluated in strata according to baseline alcohol intake (<1 g/day, >1–8 g/day, >8 g/day). Furthermore, we adjusted Model 2 for the baseline alcohol intake (<1 g/day, >1–8 g/day, >8 g/day) to investigate whether an association between a change in alcohol intake and risk of BC was independent of participants’ initial alcohol intake. We assessed estimates of Model 2 according to the time occurring between the baseline and follow-up assessments (≤5y, >5 to 10y, and >10y) to examine whether the time during which a change in alcohol consumption could occur influenced the results. Estimates of Model 2 were also computed separately by age at the follow-up assessment (<65y and ≥65y) to examine whether changes in alcohol consumption implemented earlier in life had a greater impact on the risk of BC compared to changes implemented at older ages. Furthermore, perimenopausal participants (n = 15,026) who went through menopause during the time of follow-up were excluded to investigate whether the menopausal transition that is characterised by significant hormonal changes influenced the results.

To investigate if the influence of abstainers and potentially former drinkers whose abstinence could be due to health issues affected the association [23], we excluded participants with an alcohol intake <1 g/day at both assessments. In addition, to investigate the risk of reverse causation induced by health-related issues possibly related to BC (i.e. sick-quitters), we omitted all participants diagnosed with BC within the first 2 years of follow-up. In addition to analysing change between categories of alcohol intake using stable intake as the reference, we conducted an analysis using low alcohol consumption (≤1 g/day) at both assessments as reference category. Finally, we conducted the main analysis of change in alcohol consumption modelled using complete case data.

All statistical tests were two-sided and a *p*-value of <0.05 was considered statistically significant. All analyses were conducted in R version 4.1.2. (R Foundation for Statistical Computing, Vienna, Austria) [24].

## Results

### Participant characteristics

The median time from recruitment to the follow-up assessment was 9.8 years (10–90th percentiles: 3.6–12.4). Figure 2 shows transitions in alcohol consumption categories between the two alcohol assessments among participants with complete information about their alcohol intake (n = 120,542). Across the two assessments, most participants within each category of alcohol intake (≤1 g/day, >1–8 g/day, and >8 g/day) sustained a stable alcohol intake (72.3%, 49.6%, and 73.3%, respectively).

In Table 1, characteristics of participants according to changes in alcohol intake: “decreased intake” (<−8 g/day and −8 to <−1 g/day); “stable intake” (−1 to 1 g/day); “increased intake” (1 to 8 g/day and >8 g/day) and “participants missing alcohol intake at follow-up” are shown. Participants who either decreased, increased their alcohol intake or had missing information about follow-up alcohol intake were slightly younger at baseline compared to those with stable intake. At follow-up, participants with missing data about their alcohol intake were slightly older compared to participants in the other categories of intake. Participants who changed their alcohol intake in either direction tended to have higher educational levels compared to those with stable intake or missing follow-up alcohol data. Participants with a stable intake or missing follow-up alcohol intake had a higher BMI. Online Resource, Supplementary Table S4 shows characteristics of participants with complete information.

### Continuous changes and BC risk

During a median follow-up time of 4.0 years (IQR: 2.9–8.7 years), 2,173 participants were diagnosed with BC. Modelling changes in alcohol consumption using a restricted cubic spline, we found no indication of non-linearity, *p* > 0.05 (Online Resource, Supplementary Fig. S2 using complete-case data and Online Resource, Supplementary Fig. S3). We found no association between a 10 g/day change in alcohol consumption and risk of overall BC (Model 2 HR: 0.98, 95% CI 0.96–1.01; Table 2) in the multiple imputed data adjusted for potential confounders. Similar results were seen in analyses of hormonal receptor subtypes of BC (Table 2).

**Table 2:** Associations between a 10 g/day changes in alcohol intake and the risk of overall breast cancer and hormonal receptor specific subtypes of breast cancer (n = 123,679)

Outcome	Overall BC	ER−/PR−	ER+/PR−	ER+/PR+	HER2−	HER2+
	HR (95% CI)	HR (95% CI)	HR (95% CI)	HR (95% CI)	HR (95% CI)	HR (95% CI)
*10 g/day change in alcohol consumption*						
Cases, n	2,173	237	244	1,023	1,159	262
Model 1	0.97 (0.93–1.01)	0.96 (0.85–1.09)	0.98 (0.87–1.10)	0.98 (0.93–1.04)	0.98 (0.93–1.03)	1.01 (0.90–1.13)
Model 2	0.97 (0.93–1.01)	0.96 (0.85–1.09)	0.98 (0.87–1.10)	0.98 (0.93–1.04)	0.98 (0.93–1.04)	1.01 (0.90–1.14)

Model 1 was adjusted for participant age (underlying timescale) and stratified by age at follow-up (in 1-year categories) and study center

Model 2 was further adjusted for educational level (none, primary, technical or professional school, secondary, school, university degree), BMI (<18.5 kg/m^2^, 18.5–24.9 kg/m^2^, 25–<30 kg/m^2^, ≥30 kg/m^2^), smoking status (current, former, never), physical activity level (inactive, moderately inactive, moderately active, active), number of full-term pregnancies/age at first full-term pregnancy (no full-term pregnancy; 1, <30 years; 1, ≥30 years; 2, <30 years; 2, ≥30 years, ≥3, <30 years; ≥3, ≥30 years), and ever use of hormonal replacement therapy (yes, no)

95%CI: 95% confidence interval, HR: Hazard ratio, g/day: gram per day; BC: Breast cancer, ER: Estrogen receptor status; PR: Progesterone receptor status; HER2: Human epidermal growth factor receptor 2, n/N: number

### Risk of invasive breast cancer according to categorical changes in alcohol consumption

Table 3 shows HRs and corresponding 95% CIs of invasive BC estimated using the multiple imputed data for combinations of changes in alcohol consumption compared to a stable intake within each intake category across baseline and follow-up. No associations were observed except when investigating participants with a modest alcohol intake at baseline. Compared to a consistent stable modest alcohol intake, a decreased alcohol intake from modest to low alcohol consumption was associated with a higher risk of BC (HR: 1.22, 95% CI 1.02–1.47). The corresponding analysis on complete case data was similar but not statistically significant (HR: 1.20, 95% CI 0.98–1.48).

**Table 3:**
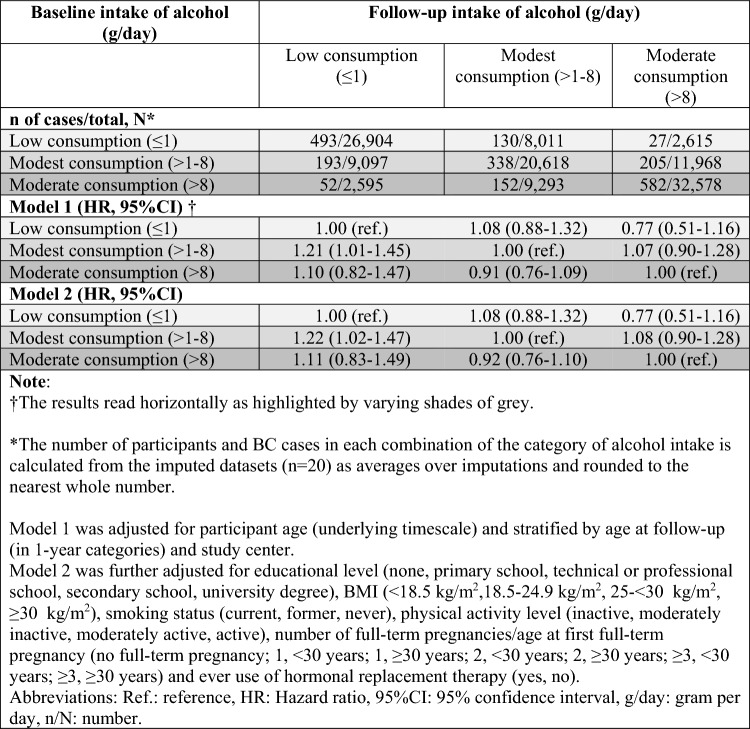
Associations between changes in alcohol intake categories and the risk of breast cancer compared to a stable intake at a given level (n = 123,679)

### Sensitivity analyses

We found no association between a 10 g/day change in alcohol consumption and risk of overall BC in any sensitivity analysis (Table 4 and Online Resource, Supplementary Table S5), which was similar to the results of the main analysis. The HR estimates and corresponding 95% CIs computed on the multiple imputed data were comparable to the estimates obtained using the complete case data (Online Resource, Supplementary Tables S6, S7, S8 and S9, respectively).

**Table 4:** Sensitivity analyses of a 10 g/day change in alcohol intake and the risk of breast cancer (n = 123,679)

Per 10 g/day change in alcohol consumption (n, cases/total)†	Overall BC (HR, 95% CI)
Adjusted for baseline intake of alcohol (≤ 1 g/day, > 1–8 g/day, > 8 g/day)	0.98 (0.94–1.02)
Baseline intake of alcohol	
≤1 g/day (650/37,529)	0.81 (0.64–1.04)
>1–8 g/day (737/41,684)	0.98 (0.88–1.09)
>8 g/day (786/44,466)	0.97 (0.92–1.02)
Time between the baseline and follow-up assessment	
≤5y (599/25,679)	0.97 (0.87–1.07)
>5–<10y (1,019/39,617)	0.96 (0.90–1.02)
>10y (555/58,383)	0.98 (0.92–1.05)
Age at the follow-up assessment	
<65y (1,506/83,759)	0.98 (0.94–1.03)
≥65y (667/39,920)	0.93 (0.87–1.01)
Excluding abstainers (<1 g/day) at both assessments (1262/73,003)^a^	0.98 (0.94–1.03)
Excluding BC cases diagnosed within first 2 years of follow-up (1413/122,919)^b^	0.98 (0.93–1.04)
Excluding perimenopausal women (1,855/108,653)^c^	0.97 (0.93–1.02)

Model 2 was adjusted for participant age (underlying timescale), educational level (none, primary school, technical or professional school, secondary, school, university degree), BMI (<18.5 kg/m^2^, 18.5–24.9 kg/m^2^, 25–<30 kg/m^2^, ≥30 kg/m^2^), smoking status (current, former, never), physical activity level (inactive, moderately inactive, moderately active, active), number of full-term pregnancies/age at first full-term pregnancy (no full-term pregnancy; 1, <30 years; 1, ≥30 years; 2, <30 years; 2, ≥30 years; ≥3, <30 years; ≥3, ≥30 years), ever use of hormonal placement therapy (yes, no) and stratified by age at follow-up (in 1-year categories) and study center

HR: Hazard ratio, 95% CI: 95% confidence interval, y: years, BC: breast cancer, g/day: gram per day, n/N: number

^†^All analyses were based on Model 2 examining associations between continuous changes in alcohol intake (10 g/day) and overall BC

^a^The number of participants included in the analysis after the exclusion of abstainers corresponds to approximately 59.0% of the total population (n = 123,679)

^b^The number of BC cases in the analysis after cases diagnosed within the first 2 years of follow-up corresponds to approximately 65.0% of the total number of cases (n = 2,173)

^c^The number of participants included in the analysis after perimenopausal participants at the time of the follow-up assessment were excluded corresponds to approximately 87.9% of the total study population (n = 123,679)

## Discussion

In this study, longitudinal data on alcohol intake was used to investigate whether changes in alcohol consumption in midlife were associated with the subsequent risk of postmenopausal BC. We found no association between a change in alcohol consumption when modeled as a continuous variable, and risk of overall BC nor hormonal subtypes of BC. In the analyses of change in categories of alcohol intake, we found some evidence of a higher risk of BC for a reduced alcohol intake from modest to low levels compared to a stable modest alcohol intake across both alcohol assessments (HR: 1.22, 95% CI 1.02–1.47). This may, however, be due to reverse causation because of subclinical disease, residual confounding, or a chance finding, as neither main results nor sensitivity analyses showed convincing evidence of associations.

### Comparison with available studies on changes in alcohol consumption and the risk of breast cancer

The knowledge about consumption of alcohol and risk of BC is extensive [2]. In contrast, the knowledge on how changes in alcohol consumption measured by consecutive assessments of participants’ alcohol intakes are associated with future risk of BC is sparse. Currently, two prospective cohort studies have investigated associations between changes in alcohol consumption as a single risk factor and the risk of BC [10, 11], while two studies have investigated changes in alcohol consumption as a part of a lifestyle index in relation to BC risk [25, 26]. Furthermore, a number of both cohort and case–control studies have examined cessation of alcohol consumption on the risk of BC based on a single assessment of the participants’ alcohol intake by comparing participants stated to be former drinkers with current drinkers. These studies have recently been reviewed in the IARC handbook of reduction or cessation of alcohol drinking (10). In a study of more than 2 million Korean women (aged ≥ 40 years) investigating changes in alcohol consumption over 2 years, a “mild intake” of alcohol (<15 g/day) at the first assessment that was reduced to a “none intake” (0 g/day) at the time of the second assessment was associated with a lower risk of BC compared to consistent mild intake [10]. Furthermore, a “heavy intake” of alcohol (≥30 g/day) that was reduced to a “moderate intake” (15.0–29.9 g/day) was associated with a lower risk of BC compared to a consistent heavy alcohol intake [10]. In a Danish cohort study of 5-year changes in alcohol intake, including 21,553 postmenopausal women, an increase in alcohol from 7–13 standard drinks/week to ≥14 standard drinks/week was associated with a higher risk of BC compared to a stable alcohol intake (<7 drinks/week). A similar result was reported for a change from <7 drinks/week to 7–13 drinks/week. However, a decrease in alcohol intake was not associated with BC when compared to a stable intake [11]. Comparing the results of these studies to ours is challenging due to differences in how alcohol consumption was operationalized. Nevertheless, the findings do not align, as we observed no association between changes in alcohol consumption and the risk of BC. In studies examining changes in alcohol intake as a part of changes in overall lifestyle, results are mixed. In a Norwegian study investigating changes in lifestyle habits using a healthy lifestyle index over an average of 7 years, a 1-unit increase in alcohol score reflecting a reduction in intake was associated with a borderline statistically significantly lower risk of BC [26]. In contrast, in a recent Swedish cohort study investigating a 12-year change in lifestyle and the risk of various cancers, no association between changes in alcohol consumption and BC incidence was observed [25]. To our knowledge, no study to date has investigated the associations between changes in alcohol consumption and the risk of BC subtypes.

### Underlying biological mechanisms

The biological mechanisms of alcohol-induced BC are not fully understood. However, consumption of alcoholic beverages has been suggested to play a role in relation to BC in early adulthood due to breast growth [27]. If this is the case, it is possible that our null findings could be explained by the fact that the changes in alcohol consumption habits took place relatively late in adulthood, and thus not in the relevant exposure window. Future studies should investigate alcohol changes at different ages, including early adulthood, in relation to subsequent risk of BC. Furthermore, reduced consumption of alcohol limits the exposure to the metabolite acetaldehyde that has DNA-damaging effects. However, while the role of acetaldehyde in head and neck carcinogenesis is well-described, its role in BC is less clear [9]. Alcohol consumption has been shown to increase circulating female sex hormones such as estrogen among postmenopausal women, which may play a role in breast carcinogenesis [28, 29]. Although the effect of cessation of alcohol consumption on sex hormones is less clear, a reduced alcohol intake may have a positive effect on plasma estrogen levels leading to a lower risk of BC [9].

### Strengths and limitations

This study has several strengths. It is based on data from a prospective large European cohort from different countries with two longitudinal measurements of alcohol intake. This enables investigations of temporal changes in alcohol consumption during midlife in a study population with different underlying drinking habits [30]. Only a limited number of studies have investigated associations between changes in alcohol consumption and risk of BC. This study contributes to a better understanding of changes in alcohol consumption in relation to BC. Other strengths include the limited loss to follow-up and the ability to investigate both overall and hormonal receptor-specific BC.

However, the study also has limitations. Questionnaire data from the follow-up assessment were not available for 40,299 women. We do not have information on the reasons for non-participation in the follow-up assessment but given that this was approximately 9.8 years after recruitment, we assume that most women were alive at this point but not available for participation. If non-participation was related to both changes in alcohol intake and diagnosis of BC, this could introduce selection bias, although the proportion of participants lost is low. Online Resource, Supplementary Table S2 shows that baseline alcohol intake was similar between participants and non-participants. However, whether non-participation was related to the incidence of BC is harder to determine based on the information available. Furthermore, a large proportion of potential eligible participants from study centers in Denmark, the Netherlands and Italy were excluded from the study as their follow-up alcohol intake was not centralized in EPIC (n = 52,334). Exclusion of these women may have led to a lower number of BC cases, and thus statistical power, including a lower number of women with higher alcohol intake, given that Danish women have among the highest alcohol intake in EPIC [31]. In the study, perimenopausal women (n = 15,026, corresponding to approximately 12% of the study population) were grouped as postmenopausal under the assumption that these women would be postmenopausal at the time of BC diagnosis. We acknowledge that this approach could have resulted in misclassification of menopausal status, resulting in the inclusion of perimenopausal BC cases. However, it is unlikely that this misclassification was dependent on alcohol consumption levels or changes. Another important limitation of our study is the short median follow-up time (median follow-up time: 4.0, IQR: 2.9–8.7). We cannot rule out that the time after which changes in alcohol intake could have impacted the risk of BC was too short. Furthermore, changes in alcohol consumption could have occurred at any time between baseline and follow-up and may not represent long-term changes in drinking habits. This corresponds to exposure measurement error. If alcohol changes impact BC risk, we also cannot rule out that the changes in alcohol consumption in this study might have been too small to be of relevance in terms of BC incidence. However, when we excluded abstainers from the analysis, which accounted for a large part of the study population (approximately 41.0%), the results did not change. Similarly, a large proportion of participants did not change their alcohol intake between baseline and follow-up but sustained a stable intake.

Moreover, while case ascertainment methods differed across countries, stratification by study center ensures that participant comparisons were always among participants with the same probability of case detection. Although we were able to investigate hormonal receptor status-specific BC, only a relatively limited number of BC cases had information about hormonal receptor status. Similarly, we did not have enough BC cases with triple-negative BC (ER−/PR−/HER2−) (cases, n = 133) to investigate associations between changes in alcohol consumption and this subtype. We therefore cannot rule out that out we had limited statistical power to detect an association between changes in alcohol consumption and risk of hormonal receptor specific BC.

Although the dietary questionnaires used to assess the participants’ alcohol intake in EPIC were validated [32], the estimated changes in alcohol consumption habits across the two assessment times were based on self-reports of alcohol intakes and therefore prone to measurement errors. We find the risk of differential measurement error regarding the exposure to be unlikely because of the prospective design of the study. Further, if non-differential intra-individual measurement errors of the exposure are similar at the baseline and follow-up assessment, the difference between these two measures should capture changes in alcohol consumption without bias. We used data from baseline to adjust for confounding to mitigate exposure-confounder feedback. However, we cannot rule out that residual confounding remains.

## Conclusion

In this study, changes in alcohol consumption during middle adulthood were not associated with the risk of invasive postmenopausal BC over a median follow-up of 4 years. However, we cannot rule out the null findings were caused by limited power due a low number of cases given the short follow-up time, in particular in the analyses of hormonal subtype specific BC.

## Supplementary Information

Below is the link to the electronic supplementary material.

Supplementary file1 (DOC 647 kb)

## Data Availability

Data requests must be directed to the corresponding author.

## Declarations

### Generative AI

Generative artificial intelligence (ChatGPT and Microsoft Copilot) was used for language refinement and proof reading of parts of the manuscript as well as to refine R code for the statistical analysis. All suggestions were evaluated by the authors.
