# Supplementary material for: Changes in alcohol consumption and the risk of postmenopausal breast cancer in the European Prospective Investigation into Cancer and Nutrition cohort
Source: Eur J Nutr. 2026 Jun 19;65(5):170. doi: 10.1007/s00394-026-04008-5 (PMC13282222; doi:10.1007/s00394-026-04008-5)
Supplement: Supplementary file 1 — Supplementary file1 (DOC 647 kb) [file 394_2026_4008_MOESM1_ESM.docx]

**Supplementary materials: Changes in alcohol consumption and the risk of postmenopausal breast cancer in the European Prospective Investigation into Cancer and Nutrition cohort**

**Journal: European Journal of Nutrition**

Christian S. Antoniussen^1^, Daniel B. Ibsen^1,2,3^, Anja Olsen^1,4^, Kim Overvad^1^, Fanélie Vasson^5^, Gianluca Severi^6,7^, Dzevka Dragic^6^, Thérèse Truong^6^, Renée T. Fortner^8,9^, Charlotte Le Cornet^8^, Matthias B. Schulze^10,11^, Chiara Di Girolamo^12^, Valeria Pala^13^, Chiara Doccioli^14^, Antonio Agudo^15,16^, Marcela Guevara^17,18,19^, Sander Tin Tin^20^, Isobel G. Jackson^21^, Marc J. Gunter^21^, Laure Dossus^5^, Pietro Ferrari^5^, Christina C. Dahm^1^

Kim Overvad passed away before submission of the paper.

Pietro Ferrari and Christina C. Dahm contributed equally to this work.

**Corresponding author:**

Christina C. Dahm

ccd@ph.au.dk

Department of Public Health, Aarhus University

Bartholins Allé 2, DK-8000 Aarhus C

ORCID identifier: https://orcid.org/0000-0003-0481-2893

**Supplementary Table 1: Number of participants with and without a follow-up assessment according to country**

| **Number of participants with and without a follow-up assessment, respectively** | | |
| --- | --- | --- |
| **Country** | **Participants with a follow-up assessment (n)** | **Participants without a follow-up assessment (n)** |
| France | 48,653 | 10,953 |
| Italy | 16,924 | 4,307 |
| Spain | 24,287 | 326 |
| United Kingdom | 33,720 | 17,709 |
| The Netherlands | 6,214 | 4,946 |
| Germany | 24,283 | 2,058 |
| **Total** | 154,081 | 40,299 |
| Abbreviations: n: numbers. | | |

**Supplementary Table 2: Baseline characteristics of participants included in the study and participants non-eligible due to no follow-up assessment.**

|  | **Participants included in the study (n=123,679)†** | **Participants without an follow-up assessment (n=40,299)** |
| --- | --- | --- |
| **Baseline characteristics**^1^ |  | |
| Age at baseline (y), median (p10-p90)  *Missing* | 52.2 (44.1, 63.0)  0 | 48.8 (28.1, 65.0)  0 |
| Alcohol intake (g/day), median (p10-p90)  *Missing* | 4.1 (0.0, 24.2)  0 | 4.3 (0.0, 24.0)  0 |
| Educational level, n (%)  None  Primary  Technical/professional school  Secondary school  University degree  *Missing* | 7,607 (6.2)  26,279 (21.2)  17,819 (14.4)  32,451 (26.2)  31,622 (25.6)  7,901 (6.4) | 415 (1.0)  7,665 (19.0)  7,757 (19.2)  9,935 (24.7)  10,830 (26.9)  3,697 (9.2) |
| BMI, n (%)  <18.5 kg/m^2^  18.5-24.9 kg/m^2^  25-<30 kg/m^2^  ≥30 kg/m^2^  *Missing* | 2,643 (2.1)  71,554 (57.9)  34,401 (27.8)  15,081 (12.2)  0 | 1,183 (2.9)  24,357 (60.4)  10,554 (26.2)  4,205 (10.4)  0 |
| Smoking status, n (%)  Never  Former  Current  *Missing* | 79,377 (64.2)  26,412 (21.4)  14,714 (11.9)  3,176 (2.6) | 22,382 (55.5)  9,359 (23.2)  7,446 (18.5)  1,112 (2.8) |
| Physical activity level, n (%)  Inactive  Moderately inactive  Moderately active  Active  *Missing* | 29,527 (23.9)  47,638 (38.5)  30,857 (24.9)  14,077 (11.4)  1,580 (1.3) | 9,703 (24.1)  14,009 (34.8)  9,123 (22.6)  5,861 (14.5)  1,603 (4.0) |
| Number of full-term pregnancies/age at first full term pregnancy, n (%)  No full-term pregnancy  1, <30 y  1, ≥30 y  2, <30 y  2, ≥30 y  ≥3, <30 y  ≥3, ≥30 y  *Missing* | 14,456 (11.7)  13,994 (11.3)  6,006 (4.9)  42,629 (34.5)  6,096 (4.9)  30,786 (24.9)  1,833 (1.5)  7,879 (6.4) | 10,527 (26.1)  3,925 (9.7)  1,981 (4.9)  9,715 (24.1)  1,619 (4.0)  6,920 (17.2)  403 (1.0)  5,209 (12.9) |
| Ever use of hormonal replacement therapy, n (%)  Yes  No  *Missing* | 35,589 (28.8)  80,520 (65.1)  7,570 (6.1) | 8,070 (20.0)  30,845 (76.5)  1,384 (3.4) |
| **Note**:  †Participants considered postmenopausal at the follow-up assessment.  ^1^Numbers are rounded to one decimal place.  Abbreviations: n/N: Number, p: Percentile, %: Percentage, BMI: Body Mass Index, g/day: gram per day, y: years. | | |

**Supplementary Table 3: Number of participants with available information about their alcohol intake at baseline and follow-up (irrespective of missing covariate information), median intake, and median time between the baseline and follow assessments according to country**

| **Country^1^** | **n, baseline/N, total (%)** | **Alcohol intake at baseline (g/day)*** | **n, follow-up/N, total (%)** | **Alcohol intake at follow-up (g/day)*** | **Time difference†*** |
| --- | --- | --- | --- | --- | --- |
| **France** | 48,270/48,720 (100) | 5.8 (0.0-28.4) | 47,721/48,720 (97,9) | 7.7 (0.0-30.3) | 12.0 (11.8-12.1) |
| **Italy** | 15,703/15,703 (100) | 3.6 (0.0-24.8) | 13,847/15,703 (88.2) | 1.9 (0.0-17.8) | 11.2 (9.6-12.6) |
| **Spain** | 14,001/14,001 (100) | 0.0 (0.0-13.5) | 14,001/14,001 (100) | 0.0 (0.0-7.7 | 3.2 (3.0-3.5) |
| **United Kingdom** | 22,889/22,889 (100) | 3.3 (0.4-16.0) | 22,711/22,889 (99.2) | 5.9 (0.0-27.5) | 5.5 (5.1-7.0) |
| **The Netherlands** | 4,458/4,458 (100) | 4.3 (0.0-24.7) | 3,978/4,458 (89.2) | 7.4 (0.0-28.3) | 10.0 (9.5-10.5) |
| **Germany** | 18,358/18,358 (100) | 5.1 (0.5-23.4) | 18,284/18,358 (99.6) | 3.3 (0.3-19.5) | 5.2 (4.1-5.7) |
| **Total** | 123,679/123,679 (100) | - | 120,542/123,679 (97.5) | - | - |
| **Note**:  ^1^Numbers are rounded to the first decimal place, *Median (interquartile range), †: Time difference in years between the baseline and follow-up (median (interquartile range) assessments for all participants irrespectively of missing information about covariates and alcohol intake at follow-up.  Abbreviations: %: Percentage, g/day: gram per day, n/N; number of participants with available information/total number of participants in the study from the specific country. | | | | | |

**Supplementary Fig. 1: Directed Acyclic Graph (DAG) used to select potential confounders**

**Unadjusted model**:


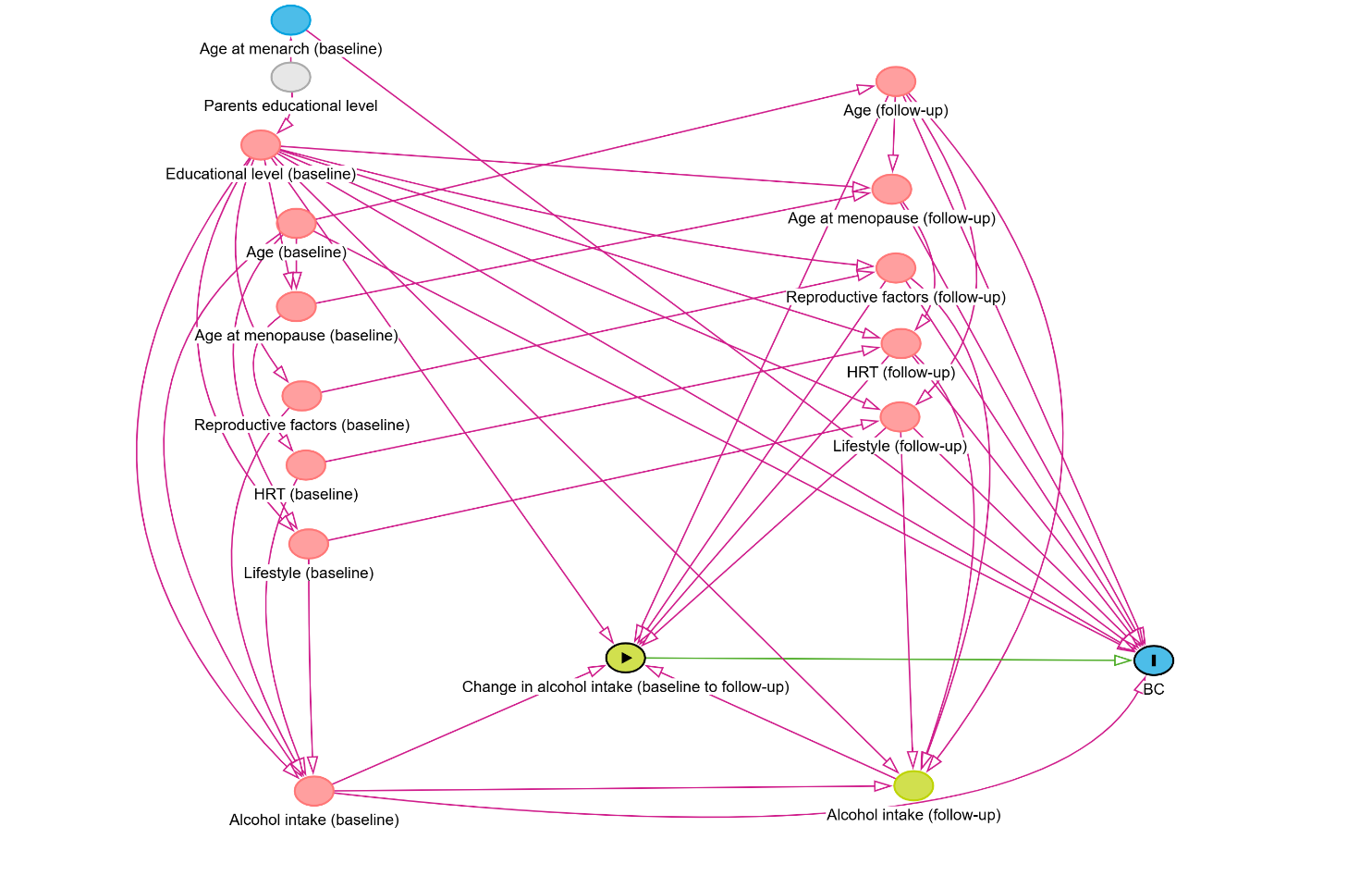


**Adjusted model**:


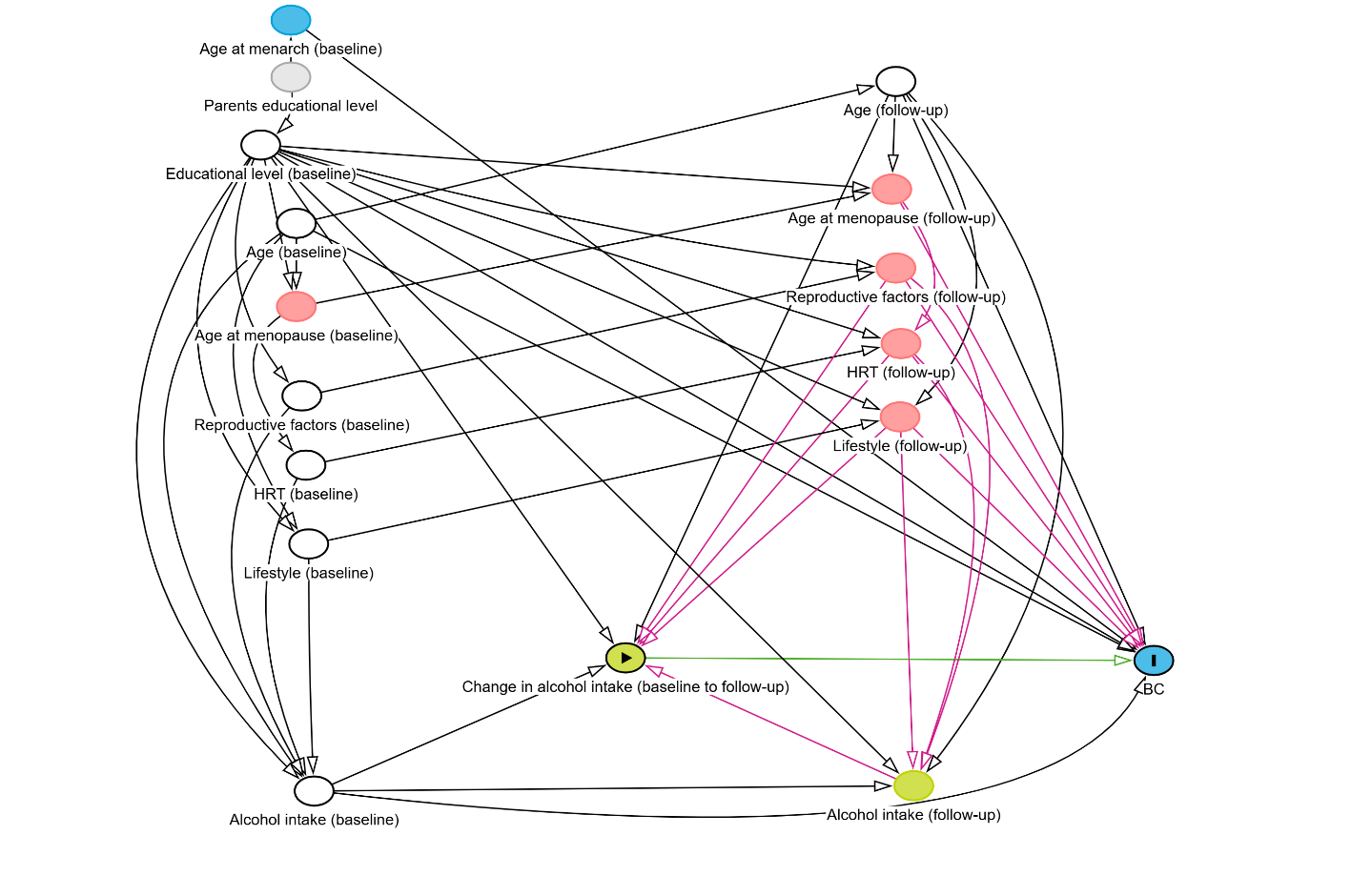


**Note**: The DAGs are kept in a simplified form to maintain readability. The variable “Educational level” is considered a proxy for socioeconomic status. The “lifestyle” variable includes “smoking status”, “physical activity level” and “BMI”. The reproductive factor variable includes “number of full-term pregnancies” and “age at first full-term pregnancy”. The “HRT” variable represents hormonal replacement therapy. The white circles in the adjusted model represents variables that were adjusted for in the analysis. We adjusted for covariates measured at baseline only to avoid exposure-confounder feedback. Age is adjusted for as the underlying timescale. “Alcohol intake at baseline” represents the alcohol intake at recruitment into the study, while “Alcohol intake at follow-up” represents the alcohol intake at the follow-up assessment. “Alcohol intake at baseline” was adjusted for in a sensitivity analysis. The DAGs were created using the online resource “dagitty” [1].

**Supplementary Table 4: Characteristics of participants with complete information on covariates according to continuous changes in alcohol consumption between the baseline and follow-up assessments (n=96,970)**

| **Continuous change in intake of alcohol (g/day) between baseline and follow-up (n=96,970)** | | | | | |
| --- | --- | --- | --- | --- | --- |
|  | **Decreased intake** | | **Stable intake** | **Increased intake** | |
| **Characteristics^1^** | **<-8** | **>-8 to <-1** | **-1 to 1** | **>1 to 8** | **>8** |
| Number of participants, n (%) | 11,554 (11.9) | 20,719 (21.4) | 31,126 (32.1) | 20,610 (21.3) | 12,961 (13.4) |
| Number of BC cases, n (%) | 204 (1.8) | 347 (1.7) | 545 (1.8) | 374 (1.8) | 207 (1.6) |
| Estrogen receptor status (ER), n (%)†  ER-  ER+  *Missing** | 23 (11.3)  153 (75.0)  28 (13.7) | 53 (15.3)  229 (66.0)  65 (18.7) | 59 (10.8)  385 (70.6)  101 (18.5) | 50 (13.4)  249 (66.6)  75 (20.1) | 23 (11.1)  153 (73.9)  31 (15.0) |
| Progesterone receptor status (PR), n (%)†  PR-  PR+  *Missing** | 49 (24.0)  116 (56.9)  39 (19.1) | 93 (26.8)  162 (46.7)  92 (26.5) | 110 (20.2)  285 (52.3)  150 (27.5) | 89 (23.8)  154 (41.2)  131 (35.0) | 41 (19.8)  94 (45.4)  72 (34.8) |
| Human epidermal growth factor receptor 2 (HER2), n (%)†  HER2-  HER2+  *Missing** | 125 (61.3)  22 (10.8)  57 (27.9) | 184 (53.0)  45 (13.0)  118 (34.0) | 280 (51.4)  76 (13.9)  189 (34.7) | 191 (51.1)  42 (11.2)  141 (37.7) | 112 (54.1)  22 (10.6)  73 (35.3) |
| Age at baseline (y), median (p10-p90) | 52.5 (44.5, 62.2) | 53.2 (44.7, 62.8) | 53.6 (44.8, 63.2) | 51.7 (44.5, 62.4) | 51.8 (44.6, 62.6) |
| Age at follow-up (y), median (p10-p90) | 61.9 (53.2, 72.1) | 61.6 (53.0, 71.8) | 61.3 (52.1, 71.5) | 61.0 (53.0, 71.7) | 61.7 (54.0, 72.8) |
| Intake of alcohol at baseline (g/day), median (p10-p90) | 24.2 (12.0, 49.9) | 7.0 (1.9, 22.3) | 0.2 (0.0, 5.6) | 3.3 (0.0, 15.1) | 7.2 (0.4, 24.0) |
| Intake of alcohol at follow-up (g/day), median (p10-p90) | 7.7 (0.0, 25.9) | 3.1 (0.0, 17.3) | 0.0 (0.0, 5.6) | 7.7 (2.2, 19.6) | 23.9 (12.0, 51.3) |
| Educational level, n (%)  None  Primary  Technical/professional school  Secondary school  University degree | 595 (5.1)  2,878 (24.9)  1,348 (11.7)  3,423 (29.6)  3,310 (28.6) | 1,086 (5.2)  5,051 (24.4)  3,288 (15.9)  5,723 (27.6)  5,571 (26.9) | 4,694 (15.1)  9,050 (29.1)  4,033 (13.0)  7,106 (22.8)  6,243 (20.1) | 807 (3.9)  3,628 (17.6)  2,983 (14.5)  6,685 (32.4)  6,507 (31.6) | 148 (1.1)  2,026 (15.6)  1,745 (13.5)  4,616 (35.6)  4,426 (34.1) |
| BMI, n (%)  <18.5 kg/m^2^  18.5-24.9 kg/m^2^  25-<30 kg/m^2^  ≥30 kg/m^2^ | 198 (1.7)  6,826 (59.1)  3,296 (28.5)  1,234 (10.7) | 350 (1.7)  11,893 (57.4)  5,998 (28.9)  2,478 (12.0) | 703 (2.3)  14,754 (47.4)  9,595 (30.8)  6,074 (19.5) | 534 (2.6)  13,278 (64.4)  5,100 (24.7)  1,698 (8.2) | 344 (2.7)  8,951 (69.1)  2,893 (22.3)  773 (6.0) |
| Smoking status, n (%)  Never  Former  Current | 6,954 (60.2)  2,682 (23.2)  1,918 (16.6) | 13,983 (67.5)  4,345 (21.0)  2,391 (11.5) | 23,370 (75.1)  4,755 (15.3)  3,001 (9.6) | 14,120 (68.5)  4,616 (22.4)  1,874 (9.1) | 7,910 (61.0)  3,473 (26.8)  1,578 (12.2) |
| Physical activity level, n (%)  Inactive  Moderately inactive  Moderately active  Active | 2,708 (23.4)  4,820 (41.7)  2,754 (23.8)  1,272 (11.0) | 4,745 (22.9)  8,412 (40.6)  5,330 (25.7)  2,232 (10.8) | 10,027 (32.2)  11,874 (38.1)  6,517 (20.9)  2,708 (8.7) | 4,210 (20.4)  8,147 (39.5)  5,995 (29.1)  2,258 (11.0) | 2,456 (18.9)  5,122 (39.5)  3,796 (29.3)  1,587 (12.2) |
| Number of full-term pregnancies/age at first full-term pregnancy, n (%)  No full-term pregnancy  1, <30 years  1, ≥30 years  2, <30 years  2, ≥30 years  ≥3, <30 years  ≥3, ≥30 years | 1,545 (13.4)  1,613 (14.0)  677 (5.9)  4,211 (36.4)  593 (5.1)  2,771 (24.0)  144 (1.2) | 2,707 (13.1)  2,677 (12.9)  1,075 (5.2)  7,610 (36.7)  1,043 (5.0)  5,302 (25.6)  305 (1.5) | 3,717 (11.9)  3,597 (11.6)  1,540 (4.9)  10,674 (34.3)  1,560 (5.0)  9,480 (30.5)  558 (1.8) | 2,481 (12.0)  2,229 (10.8)  1,031 (5.0)  7,871 (38.2)  1,200 (5.8)  5,431 (26.4)  367 (1.8) | 1,553 (12.0)  1,412 (10.9)  634 (4.9)  5,022 (38.7)  726 (5.6)  3,424 (26.4)  190 (1.5) |
| Ever use of hormonal replacement therapy, n (%)  Yes  No | 3,705 (32.1)  7,849 (67.9) | 7,133 (34.4)  13,586 (65.6) | 8,701 (28.0)  22,425 (72.0) | 6,524 (31.7)  14,086 (68.3) | 4,344 (33.5)  8,617 (66.5) |
| **Note**:  ^1^Numbers are rounded to one decimal place  †Numbers cover cases of BC, *Cases with missing information about hormonal receptor status were censored in the Cox regression model.  Abbreviations: N/n: Number, p: Percentile, %: Percentage, BC; breast cancer, BMI: Body Mass Index, y: Years, g/d: gram per day, -: not applicable. | | | | | |

**Supplementary Fig. 2: Association between changes in alcohol consumption and risk of BC modelled with a restricted cubic spline with 3 knots (complete case analysis, n=96,970, cases, n=1,677)†**

**
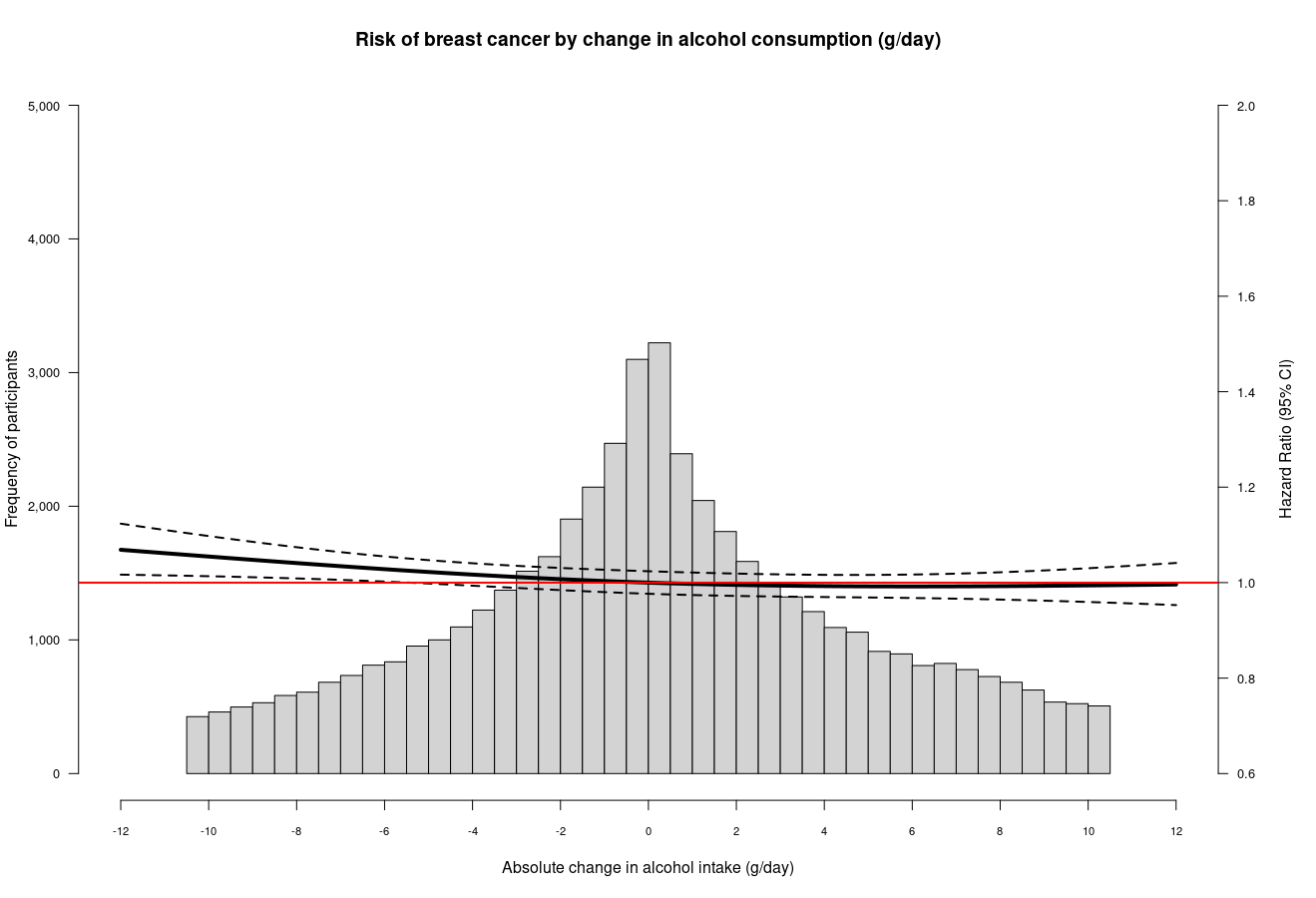
**

**Note**: †Participants with a change in alcohol consumption outside the 10.0-90.0% percentiles of the distribution of differences in alcohol intake between baseline and follow-up (corresponding to -9.5-10.5 g/day) were excluded from the plot to reduce the risk of distorting by outliers. HR estimates were adjusted for participant age (underlying timescale), educational level (none, primary school, technical or professional school, secondary school, university degree), BMI (<18.5 kg/m^2^, 18.5-24.9 kg/m^2^, 25-<30 kg/m^2^, ≥30 kg/m^2^), ever use of hormonal replacement therapy (yes, no), smoking status (current, former, never), physical activity level (inactive, moderately inactive, moderately active, active), number of full-term pregnancies/age at first full-term pregnancy (no full-term pregnancy; 1, <30 years; 1, ≥30 years; 2, <30 years; 2, ≥30 years; ≥3, <30 years; ≥3, ≥30 years and stratified by age at follow-up (in 1-year categories) and study center. Abbreviations: BC: Breast cancer, HR: Hazard ratio, 95%CI: 95% confidence interval, g/day: gram per day, N/n: number.

**Supplementary Fig. 3: Association between changes in alcohol consumption and risk of BC modelled with a restricted cubic spline with 3 knots and adjusted for baseline alcohol intake (complete case analysis, n=96,970, cases, n=1,677)†**


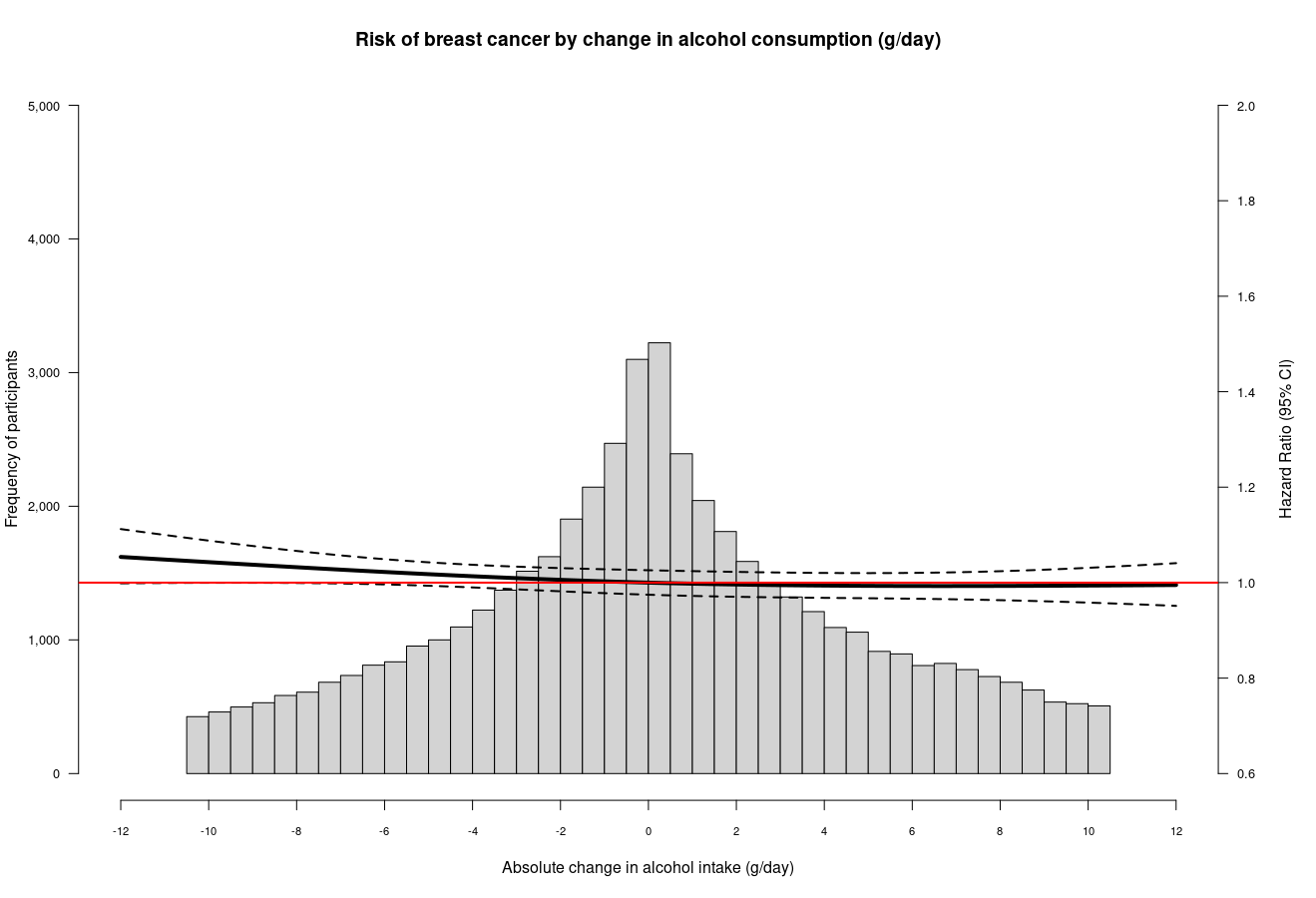


**Note**: †Participants with a change in alcohol consumption outside the 10.0-90.0% percentiles of the distribution of differences in alcohol intake between baseline and follow-up (corresponding to -9.5-10.5 g/day) were excluded from the plot to reduce the risk of distorting by outliers. HR estimates were adjusted for participant age (underlying timescale), educational level (none, primary school, technical or professional school, secondary school, university degree), BMI (<18.5 kg/m^2^, 18.5-24.9 kg/m^2^, 25-<30 kg/m^2^, ≥30 kg/m^2^), ever use of hormonal replacement therapy (yes, no), smoking status (current, former, never), physical activity level (inactive, moderately inactive, moderately active, active), number of full-term pregnancies/age at first full-term pregnancy (no full-term pregnancy; 1, <30 years; 1, ≥30 years; 2, <30 years; 2, ≥30 years; ≥3, <30 years; ≥3, ≥30 years, alcohol intake at baseline (≤1 g/day, >1-8 g/day, >8 g/day) and stratified by age at follow-up (in 1-year categories) and study center.

Abbreviations: BC: Breast cancer, HR: Hazard ratio, 95%CI: 95% confidence interval, g/day: gram per day, N/n: number.

**Supplementary Table 5: Associations between change in alcohol intake categories and risk of breast cancer compared to consistently low alcohol intake (n=123,679)**

| **Baseline intake of alcohol (g/day)** | **Follow-up intake of alcohol (g/day)** | | |
| --- | --- | --- | --- |
|  | Low consumption  (≤1) | Modest consumption (>1-8) | Moderate consumption (>8) |
| **n of cases/total, N*** | | | |
| Low consumption (≤1) | 493/26,904 | 130/8,011 | 27/2,615 |
| Modest consumption (>1-8) | 193/9,097 | 338/20,618 | 205/11,968 |
| Moderate consumption (>8) | 52/2,595 | 152/9,293 | 582/32,578 |
| **Model 1 (HR, 95%CI)** | | | |
| Low consumption (≤1) | 1.00 (ref.) | 1.08 (0.88-1.32) | 0.77 (0.51-1.16) |
| Modest consumption (>1-8) | 1.14 (0.96-1.35) | 0.94 (0.81-1.09) | 1.01 (0.85-1.20) |
| Moderate consumption (>8) | 1.26 (0.94-1.68) | 1.04 (0.86-1.26) | 1.15 (1.01-1.30) |
| **Model 2 (HR, 95%CI)** | | | |
| Low consumption (≤1) | 1.00 (ref.) | 1.08 (0.88-1.32) | 0.77 (0.51-1.16) |
| Modest consumption (>1-8) | 1.14 (0.96-1.35) | 0.93 (0.80-1.08) | 1.00 (0.84-1.19) |
| Moderate consumption (>8) | 1.24 (0.93-1.66) | 1.02 (0.85-1.24) | 1.12 (0.98-1.28) |
| **Note**: *The number of participants and BC cases in each combination of category of alcohol intake is calculated from the imputed datasets (n=20) as averages over imputations and rounded to the nearest whole number.    Model 1 was adjusted for participant age (underlying timescale) and stratified by age at follow-up (in 1-year categories) and study center.  Model 2 was further adjusted for educational level (none, primary school, technical or professional school, secondary school, university degree), BMI (<18.5 kg/m^2^,18.5-24.9 kg/m^2^, 25-<30 kg/m^2^, ≥30 kg/m^2^), smoking status (current, former, never), physical activity level (inactive, moderately inactive, moderately active, active), number of full-term pregnancies/age at first full-term pregnancy (no full-term pregnancy; 1, <30 years; 1, ≥30 years; 2, <30 years; 2, ≥30 years; ≥3, <30 years; ≥3, ≥30 years) and ever use of hormonal replacement therapy (yes, no).  Abbreviations: BC: Breast cancer, Ref.: Reference, HR: Hazard ratio, 95%CI: 95% confidence interval, g/day: gram per day, N/n: number. | | | |

**Supplementary Table 6: Associations between change in alcohol intake categories and risk of breast cancer compared to a stable intake at a given level (complete case data, n=96,970)**

| **Baseline intake of alcohol (g/day)** | **Follow-up intake of alcohol (g/day)** | | |
| --- | --- | --- | --- |
|  | Low consumption  (≤1) | Modest consumption (>1-8) | Moderate consumption (>8) |
| **n of cases/total, N** | | | |
| Low consumption (≤1) | 396/22,174 | 105/6,205 | 20/2,090 |
| Modest consumption (>1-8) | 144/7,077 | 253/15,536 | 147/9,106 |
| Moderate consumption (>8) | 42/2,199 | 127/7,286 | 443/25,297 |
| **Model 1 (HR, 95%CI)** † | | | |
| Low consumption (≤1) | 1.00 (ref.) | 1.15 (0.93-1.44) | 0.73 (0.45-1.14) |
| Modest consumption (>1-8) | 1.19 (0.97-1.46) | 1.00 (ref.) | 1.03 (0.84-1.27) |
| Moderate consumption (>8) | 1.13 (0.82-1.56) | 1.00 (0.82-1.23) | 1.00 (ref.) |
| **Model 2 (HR, 95%CI)** | | | |
| Low consumption (≤1) | 1.00 (ref.) | 1.15 (0.92-1.43) | 0.73 (0.46-1.15) |
| Modest consumption (>1-8) | 1.20 (0.98-1.48) | 1.00 (ref.) | 1.04 (0.84-1.27) |
| Moderate consumption (>8) | 1.14 (0.83-1.58) | 1.01 (0.83-1.24) | 1.00 (ref.) |
| **Note:** †The results read horizontally as highlighted by varying shades of grey.  Model 1: adjusted for participant age (underlying timescale), stratified by age at follow-up (in 1-year categories) and study center.  Model 2 was further adjusted for educational level (none/primary/technical or professional school/secondary, school/university degree), BMI (<18.5 kg/m^2^, 18.5-24.9 kg/m^2^, 25-<30 kg/m^2^, ≥30 kg/m^2^), ever use of hormonal replacement therapy (yes, no), smoking status (current, former, never), physical activity level (inactive, moderately inactive, moderately active, active), number of full-term pregnancies/age at first full-term pregnancy (No full-term pregnancy; 1, <30 years; 1, ≥30 years; 2, <30 years; 2, ≥30 years; ≥3, <30 years; ≥3, ≥30 years).  Abbreviations: Ref.: Reference, HR: Hazard ratio, 95%CI: 95% confidence interval, g/day: gram per day, N/n: number. | | | |

**Supplementary Table 7: Associations between change alcohol intake categories and risk of breast cancer compared to a consistently low alcohol intake (complete case data, n=96,970)**

| **Baseline intake of alcohol (g/day)** | **Follow-up intake of alcohol (g/day)** | | |
| --- | --- | --- | --- |
|  | Low consumption  (≤1) | Modest consumption (>1-8) | Moderate consumption (>8) |
| **n of cases/total, N** | | | |
| Low consumption (≤1) | 396/22,174 | 105/6,205 | 20/2,090 |
| Modest consumption (>1-8) | 144/7,077 | 253/15,536 | 147/9,106 |
| Moderate consumption (>8) | 42/2,199 | 127/7,286 | 443/25,297 |
| **Model 1 (HR, 95%CI)** | | | |
| Low consumption (≤1) | 1.00 (ref.) | 1.15 (0.93-1.44) | 0.73 (0.45-1.14) |
| Modest consumption (>1-8) | 1.13 (0.93-1.37) | 0.95 (0.80-1.12) | 0.98 (0.80-1.19) |
| Moderate consumption (>8) | 1.25 (0.91-1.73) | 1.12 (0.91-1.37) | 1.11 (0.96-1.29) |
| **Model 2 (HR, 95%CI)** | | | |
| Low consumption (≤1) | 1.00 (ref.) | 1.15 (0.92-1.43) | 0.73 (0.46-1.15) |
| Modest consumption (>1-8) | 1.12 (0.92-1.37) | 0.93 (0.79-1.11) | 0.97 (0.79-1.18) |
| Moderate consumption (>8) | 1.23 (0.90-1.70) | 1.09 (0.89-1.35) | 1.08 (0.93-1.26) |
| **Note**:  Model 1: adjusted for participant age (underlying timescale), stratified by age at follow-up (in 1-year categories) and study center.  Model 2 was further adjusted for educational level (none, primary school, technical or professional school, secondary school, university degree), BMI (<18.5 kg/m^2^,18.5-24.9 kg/m^2^,25-<30 kg/m^2^, ≥30 kg/m^2^), ever use of hormonal replacement therapy (yes, no), smoking status (current, former, never), physical activity level (inactive, moderately inactive, moderately active, active), number of full-term pregnancies/age at first full-term pregnancy (No full-term pregnancy; 1, <30 years; 1, ≥30 years; 2, <30 years; 2, ≥30 years; ≥3, <30 years; ≥3, ≥30 years).    Abbreviations: Ref.: Reference, HR: Hazard ratio, 95%CI: 95% confidence interval, g/day: gram per day, N/n: number. | | | |

**Supplementary Table 8: Associations between changes in alcohol consumption and risk of overall breast cancer (complete case data, n= 96,970)**

| **Per 10 g/day change in alcohol consumption** | **Overall BC** HR (95% CI) |
| --- | --- |
| **Cases, n** | 1,677 |
| **Model 1** | 0.97 (0.92-1.01) |
| **Model 2** | 0.97 (0.93-1.02) |
| **Note**:  Model 1: adjusted for participant age (underlying timescale), stratified by age at follow-up (in 1-year categories) and study center.  Model 2 was further adjusted for educational level (none/primary/technical or professional school/secondary, school/university degree), BMI (<18.5 kg/m^2^,18.5-24.9 kg/m^2^, 25-<30 kg/m^2^, ≥30 kg/m^2^), ever use of hormonal replacement therapy (yes, no), smoking status (current, former, never), physical activity level (inactive, moderately inactive, moderately active, active), number of full-term pregnancies/age at first full-term pregnancy (No full-term pregnancy; 1, <30 years; 1, ≥30 years; 2, <30 years; 2, ≥30 years; ≥3, <30 years; ≥3, ≥30 years).  Abbreviations: BC: breast cancer, HR: Hazard ratio, 95%CI: 95% confidence interval, g/day: gram per day, N/n: number. | |

**Supplementary Table 9: Sensitivity analyses of changes in alcohol intake (10 g/day) at risk of breast cancer (complete case data, n= 96,970)**

| **Per 10 g/day change in alcohol consumption (n, cases/total, N)†** | **Overall BC** HR (95% CI) |
| --- | --- |
| **Adjusted for baseline alcohol intake^a^** | 0.98 (0.93-1.02) |
| **Baseline intake of alcohol**  ≤1 g/day (521/30,469)  >1-8 g/day (544/31,719)  >8 g/day (612/34,782) | 0.77 (0.58-1.01)  1.00 (0.88-1.12)  0.97 (0.92-1.02) |
| **Time between the baseline and follow-up assessments**  ≤5y (514/21,768)  >5-<10y (713/27,083)  >10y^b^ (450/48,119) | 0.92 (0.82-1.02)  0.98 (0.91-1.05)  0.98 (0.91-1.05) |
| **Age at the follow-up assessment**  <65y (1,172/65,121)  ≥65y (505/31,849) | 0.99 (0.93-1.04)  0.94 (0.86-1.02) |
| **Excluding abstainers at both assessment (<1 g/day)^c^** (970/57,259) | 0.99 (0.94-1.04) |
| **Excluding BC cases diagnosed within the first 2 year of follow-up^d^** (1,074/96,367) | 0.97 (0.91-1.03) |
| **Excluding perimenopausal women (1,498/88,843)^e^** | 0.98 (0.93-1.03) |
| **Note**: †All analyses were based on Model 2 examining associations between changes in alcohol intake continuously (10g/day) and overall BC.  Model 2 was adjusted for participant age (underlying timescale), educational level (none, primary school, technical or professional school, secondary school, university degree), BMI (<18.5 kg/m^2^,18.5-24.9 kg/m^2^, 25-<30 kg/m^2^, ≥30 kg/m^2^), smoking status (current, former, never), physical activity level (inactive, moderately inactive, moderately active, active), number of full-term pregnancies/age at first full-term pregnancy (no full-term pregnancy, 1, <30 years, 1, ≥30 years, 2, <30 years, 2, ≥30 years, ≥3, <30 years, ≥3, ≥30 years), ever use of hormonal replacement therapy (yes, no) and stratified by age at follow-up (in 1-year categories) and study center.  ^a^Further adjusted for the baseline alcohol intake in categories (≤1 g/day, >1-8 g/day, >8 g/day)  ^b^”None” and “primary school” were combined in the adjustment variable “educational level” due to a too small number of participants with no education in this analysis (n=169).  ^c^The number of participants included in the analysis after exclusion of abstainers corresponds to 59.0%.  ^d^The number participants included in the analysis after excluding BC cases diagnosed within the first 2 years of follow-up corresponds to 99.4%.  ^e^The number of participants included in the analysis after perimenopausal participants (n=8,127) at the time of the follow-up assessment were excluded corresponds to approximately 91.6% of the total study population (n=96,970)  HR: Hazard ratio, 95%CI: 95% confidence interval, g/day: gram per day, BC: breast cancer, N/n: number. | |

**References**

1. Textor J, van der Zander B, Gilthorpe MS, et al (2016) Robust causal inference using directed acyclic graphs: the R package “dagitty.” Int J Epidemiol 45:1887–1894. https://doi.org/10.1093/ije/dyw341
